# Genomic analysis of advanced breast cancer tumors from talazoparib-treated g*BRCA1/2*mut carriers in the ABRAZO study

**DOI:** 10.1038/s41523-023-00561-y

**Published:** 2023-10-06

**Authors:** Nicholas C. Turner, A. Douglas Laird, Melinda L. Telli, Hope S. Rugo, Audrey Mailliez, Johannes Ettl, Eva-Maria Grischke, Lida A. Mina, Judith Balmaña, Peter A. Fasching, Sara A. Hurvitz, Julia F. Hopkins, Lee A. Albacker, Jijumon Chelliserry, Ying Chen, Umberto Conte, Andrew M. Wardley, Mark E. Robson

**Affiliations:** 1 The Royal Marsden Hospital, The Institute of Cancer Research, London, UK; 2 Pfizer Inc., La Jolla, CA USA; 3 Stanford University School of Medicine, Stanford, CA USA; 4 University of California San Francisco Helen Diller Family Comprehensive Cancer Center, San Francisco, CA USA; 5 Department of Medical Oncology, Breast Cancer Unit, Centre Oscar Lambret, Lille, France; 6 Department of Obstetrics and Gynecology, Klinikum rechts der Isar, Technische Universität München, Munich, Germany; 7 Universitӓts Frauenklinik Tübingen, Eberhard Karls University, Tübingen, Germany; 8 Banner MD Anderson Cancer Center, Gilbert, AZ USA; 9 Hospital Vall d’Hebron, and Vall d’Hebron Institute of Oncology, Universitat Autònoma de Barcelona, Barcelona, Spain; 10 University Hospital Erlangen, Department of Gynecology and Obstetrics, Friedrich-Alexander University Erlangen-Nuremberg, Comprehensive Cancer Center Erlangen-EMN, Erlangen, Germany; 11 University of California, Los Angeles/Jonsson Comprehensive Cancer Center (UCLA/JCCC), Los Angeles, CA USA; 12 Foundation Medicine Inc., Cambridge, MA USA; 13 Pfizer Inc., New York, NY USA; 14 Manchester Breast Centre, Division of Cancer Sciences, University of Manchester, Manchester, UK; 15 Memorial Sloan Kettering Cancer Center, New York, NY USA

**Keywords:** Tumour biomarkers, Pharmacogenomics, Breast cancer

## Abstract

These analyses explore the impact of homologous recombination repair gene mutations, including *BRCA1/2* mutations and homologous recombination deficiency (HRD), on the efficacy of the poly(ADP-ribose) polymerase (PARP) inhibitor talazoparib in the open-label, two-cohort, Phase 2 ABRAZO trial in germline *BRCA1/2*-mutation carriers. In the evaluable intent-to-treat population (*N* = 60), 58 (97%) patients harbor ≥1 *BRCA1/2* mutation(s) in tumor sequencing, with 95% (53/56) concordance between germline and tumor mutations, and 85% (40/47) of evaluable patients have *BRCA* locus loss of heterozygosity indicating HRD. The most prevalent non-*BRCA* tumor mutations are *TP53* in patients with *BRCA1* mutations and *PIK3CA* in patients with *BRCA2* mutations. *BRCA1-* or *BRCA2*-mutated tumors show comparable clinical benefit within cohorts. While low patient numbers preclude correlations between HRD and efficacy, germline *BRCA1/2* mutation detection from tumor-only sequencing shows high sensitivity and non-*BRCA* genetic/genomic events do not appear to influence talazoparib sensitivity in the ABRAZO trial.

**ClinicalTrials.gov identifier:** NCT02034916.

## Introduction

The tumor suppressors *breast cancer susceptibility genes BRCA1* and *BRCA2* are critical to the repair of double-strand breaks in DNA via homologous recombination repair (HRR). During tumorigenesis, loss of the *BRCA* wildtype alleles leads to the use of other repair pathways, notably those involving poly(ADP-ribose) polymerase (PARP) 1 and 2^1,2^. PARP inhibition in *BRCA*-mutated cells that have deficient HRR results in cell death due to synthetic lethality^1,3^. Investigations have also introduced the concept of “BRCAness” where constitutional methylation of the *BRCA1* promoter^4^ or deficiencies in other HRR proteins, aside from BRCA1/2, render cells sensitive to PARP inhibitors (PARPi)^3,5–7^.

This initial model explaining PARPi efficacy based on synthetic lethality alone was modified when preclinical data showed that some PARPi trapped PARP1 on DNA in addition to PARP1 catalytic inhibition^8,9^. It is hypothesized that trapped PARP may impede replication fork machinery directly^10^ or prevent replication fork progression, resulting in damaged DNA that cannot be repaired by cells with defective HRR mechanisms^1^. Studies have shown that the degree of trapping varies between different PARPi, with talazoparib displaying the greatest potency^1,9,11^.

Clinical trials have demonstrated the efficacy of talazoparib in breast cancers with germline *BRCA1/2* mutations (g*BRCA1*/*2*mut)^12,13^. ABRAZO (NCT02034916) was a two-cohort, Phase 2 study of talazoparib in g*BRCA1/2*mut carriers with a response to prior platinum with no progression on or ≤8 weeks of the last platinum dose (Cohort 1), or ≥3 platinum-free cytotoxic regimens (Cohort 2) for advanced breast cancer. Here, talazoparib demonstrated a confirmed objective response rate (ORR) of 20.8% (95% confidence interval [CI] 10.47–34.99) and 37.1% (95% CI 21.47–55.08) in Cohorts 1 and 2, respectively^12,14^. Investigator-assessed median progression-free survival (PFS) was 4.0 months (95% CI 2.8–5.4) in Cohort 1 and 5.6 months (95% CI 5.5–7.8) in Cohort 2. An exploratory subgroup analysis suggested that a longer platinum-free interval following the last dose of platinum therapy was associated with greater clinical activity^12^.

Mutations in genes involved in HRR are associated with better outcomes after PARPi therapy in prostate cancer^15^, but it is unclear which tumor genetic or genomic factors might influence PARPi response in patients with human epidermal growth factor receptor 2-negative (HER2−), g*BRCA1/2*mut locally advanced or metastatic breast cancer (MBC). Despite studies suggesting that the inactivation or deletion of a single *BRCA1/2* allele, resulting in haploinsufficiency, can be enough to promote tumorigenesis^16,17^, patients with g*BRCA1/2*mut tumors frequently exhibit tumoral loss of non-mutated (wildtype) allele at the *BRCA1* or *BRCA2* locus, known as locus-specific loss of heterozygosity (LOH)^16–18^. Indeed, the presence of LOH has been shown to be associated with high sensitivity to PARPi^16,18^.

The goal of these analyses was to assess tumor tissue from patients enrolled in ABRAZO, with a focus on *BRCA1/2*mut, including germline-tumor concordance and zygosity; other genes implicated in homologous recombination DNA damage repair (DDR); other commonly mutated non-DDR genes; homologous recombination deficiency (HRD), assessed using genome-wide LOH (gLOH); and to explore potential correlations of the above with efficacy outcomes. Here, 97% of patients have ≥1 *BRCA1/2* mutation with 95% concordance between germline and tumor mutations. The most prevalent non-*BRCA* tumor mutations are *TP53* and *PIK3CA*. *BRCA* LOH is evident in 85% of t*BRCA*mut patients evaluable for *BRCA* zygosity and 81.6% of patients have gLOH ≥16% across both cohorts. Overall, *BRCA1*- or *BRCA2*-mutated tumors show comparable clinical benefit within cohorts while non-*BRCA* genetic/genomic events do not appear to influence talazoparib sensitivity.

## Results

### Patients

**Fig. 1:**
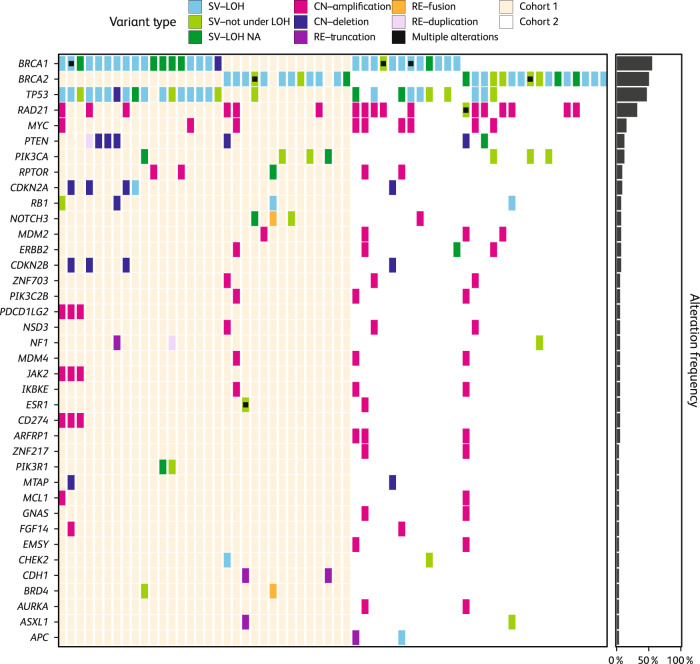
Tumor known/likely pathogenic variants detected in ABRAZO^1^. ^1^Known/likely pathogenic variants per FoundationOne^®^ CDx test are shown (genes altered in >1 patient are plotted). Those patients with multiple alterations in a gene are indicated by (■) and if one of the alterations is LOH, the square is colored as LOH. For rearrangements, if a partner gene was present, both genes were labeled. CN copy number, LOH loss of heterozygosity, NA not available, RE rearrangement, SV short variant.

A total of 84 patients enrolled between May 2014 and February 2016 comprised the intent-to-treat (ITT) population of the ABRAZO trial^12^. The median follow-up time was 13.7 months for each cohort^12^. Baseline characteristics are shown in Table 1. Tumor tissue was evaluable for sequencing from 60/84 patients (71%) with a similar number of evaluable patients in both cohorts (Table 1, Fig. 1, Supplementary Fig. 1)^19^.

**Table 1:** Summary of baseline characteristics (evaluable ITT population).

Characteristics	Cohort 1 *n* = 32	Cohort 2 *n* = 28	Total *N* = 60
Age, median (range), years	51 (31–74)	53 (33–75)	52 (31–75)
ECOG performance status = 0	21 (66)	9 (32)	30 (50)
History of CNS metastasis	3 (9)	1 (4)	4 (7)
Visceral disease	25 (78)	18 (64)	43 (72)
Hormone receptor status			
HER2-positive	0	4 (14)	4 (7)
Triple-negative	19 (59)	3 (11)	22 (37)
ER-positive or PgR-positive	13 (41)	25 (89)	38 (63)
*BRCA* mutation status			
* BRCA1*-positive	17 (53)	12 (43)	29 (48)
* BRCA2*-positive	14 (44)	16 (57)	30 (50)
Unknown	1 (3)	0	1 (2)
Number of prior cytotoxic regimens for advanced disease			
1 to 2	17 (53)	1 (4)^a^	18 (30)
3 to 4	9 (28)	16 (57)	25 (42)
≥5	6 (19)	11 (39)	17 (28)

All values are presented as the number of patients (%), unless otherwise stated.

Cohort 1 comprised patients with response to prior platinum and no progression within 8 weeks and Cohort 2 comprised patients who received ≥3 platinum-free cytotoxic regimens. Evaluable ITT population includes all the patients with tumor samples suitable for genomic evaluation and analyzed using FoundationOne^®^ CDx assay.

*BRCA* *breast cancer susceptibility gene,* *CNS* central nervous system, *ECOG* Eastern Cooperative Oncology Group, *ER* estrogen receptor, *HER2* human epidermal growth factor receptor 2, *ITT* intent-to-treat, *PgR* progesterone receptor.

^a^Protocol deviation: eligibility criteria not met (≥3 prior cytotoxic regimens).

### Prevalence and types of *BRCA1/2* mutations found in tumors

Of 60 evaluable patients, 58 (97%) exhibited ≥1 *BRCA1* or *BRCA2* pathogenic tumor mutation (t*BRCA1/2*mut); no patients had both *BRCA1* and *BRCA2* mutations (Table 2, Fig. 1). The two patients without a t*BRCA1/2*mut had *BRCA2* variants of unknown pathogenic significance distinct from their g*BRCA2*mut (Table 2, Supplementary Table 1 [patients 30 and 36]). The landscape of tumor genetic alterations in ABRAZO based on testing with FoundationOne^®^ CDx is shown in Fig. 1. The distribution of *BRCA* mutations was not uniform, with *BRCA1* mutations more commonly observed in Cohort 1 than Cohort 2. Conversely, *BRCA2* mutations were more prevalent in Cohort 2 than Cohort 1 (Table 2, Fig. 1). Across both cohorts, the most common tumor *BRCA1/2* variant types detected were single nucleotide variants (*BRCA1*: 15/60, 25.0%; *BRCA2*: 11/60, 18.3%), deletions (*BRCA1*: 11/60, 18.3%; *BRCA2:* 12/60, 20.0%), and insertions (*BRCA1*: 4/60, 6.7%; *BRCA2*: 6/60, 10.0%), with a tumor *BRCA1* copy number alteration (CNA) only evident in 1/60 patients (Supplementary Table 1 [patient 16]).

**Table 2:** Summary of tumor *BRCA1/2* mutations and loss of heterozygosity (evaluable ITT population).

Tumor *BRCA1/2* mutations and LOH	Cohort 1 *n* (%)	Cohort 2 *n* (%)	Total *N* (%)
No. of evaluable patients^a^	32	28	60
Only *BRCA1* mutation(s)^b^	18 (56.3)	12 (42.9)	30 (50.0)
Only *BRCA2* mutation(s)^c^	12 (37.5)	16 (57.1)	28 (46.7)
Both *BRCA1* and *BRCA2* mutation(s)	0 (0.0)	0 (0.0)	0 (0.0)
Neither *BRCA1* nor *BRCA2* mutation(s)^d^	2 (6.3)	0 (0.0)	2 (3.3)
No. evaluable for *BRCA* zygosity^e^	23	24	47
≥1 *BRCA1* or *BRCA2* mutation with LOH	21 (91.3)	19 (79.2)	40 (85.1)
≥1 *BRCA1* mutation with LOH	12 (52.2)	10 (41.7)	22 (46.8)
≥1 *BRCA2* mutation with LOH	9 (39.1)	9 (37.5)	18 (38.3)
No *BRCA1* or *BRCA2* mutations with LOH	2 (8.7)	5 (20.8)	7 (14.9)

Cohort 1 comprised patients with response to prior platinum and no progression within 8 weeks and Cohort 2 comprised patients who received ≥3 platinum-free cytotoxic regimens. Evaluable ITT population includes all the patients with tumor samples suitable for genomic evaluation and analyzed using FoundationOne^®^ CDx assay. One patient exhibited no known/likely pathogenic *BRCA* mutation but did exhibit a pathogenic *BRCA1* CNA per FoundationOne^®^ CDx. However, based on further examination of primary tumor sequencing data by Foundation Medicine, this CNA was deemed to align with a germline *BRCA1* del exons 13–15 mutation in the same patient, hence this subject was included in the *BRCA*mut tally for this table^19^.

*BRCA1/2* *breast cancer susceptibility gene 1 or 2,* *CNA* copy number alteration, *gBRCA1/2mut* germline *BRCA1/2* mutation, *ITT* intent-to-treat, *LOH* loss of heterozygosity, *tBRCA1/2mut* tumor BRCA1/2 mutation.

^a^The percentages are calculated by using the number of evaluable patients in each cohort or the combined total number as the denominator.

^b^Median (min, max) number of distinct *BRCA1* mutations per patient in the only *BRCA1* mutation category = 1(1,2).

^c^Median (min, max) number of distinct *BRCA2* mutations per patient in the only *BRCA2* mutation category = 1(1,2).

^d^Two patients without a t*BRCA1/2*mut had *BRCA2* variants of unknown pathogenic significance distinct from their g*BRCA2*mut: First patient: g*BRCA2*mut = 9345 G > C (P3039P) with t*BRCA2*variant = 5070 A > C (K1690N); Second patient: g*BRCA2*mut = duplicate exons 15–18 with t*BRCA2*variant = 7052 C > T (A2351V).

^e^The percentages are calculated by using the number of evaluable patients in each cohort as the denominator. LOH is predicted by somatic-germline-zygosity analysis (Foundation Medicine, Inc.). LOH can refer to either copy-neutral LOH status (i.e., homozygous, both alleles carry the same variant in the tumor) or to hemizygous status (i.e., loss of one allele in the tumor). There were no patients who exhibited mutations in both *BRCA1* and *BRCA2*.

**Fig. 2:**
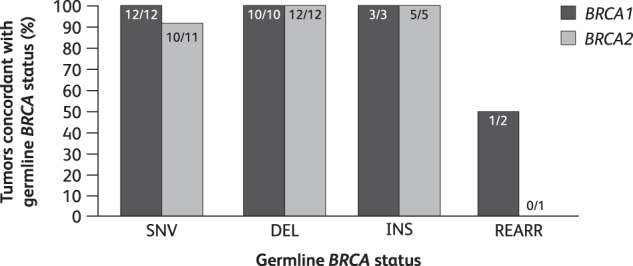
Tumor sequencing has high sensitivity for germline *BRCA1/2* mutations^1^. ^1^The proportion of patients with a known g*BRCA1*mut based on the BRACAnalysis CDx^®^ assay (Myriad Genetics) who have a *BRCA1* mutation detected in tumor using FoundationOne^®^ CDx is shown, and similarly for *BRCA2*. All patients showing concordant *BRCA1* or *BRCA2* mutational status exhibited the same mutation in tumor as originally detected in germline, as evidenced by mapping to a common Variation ID in ClinVar (https://www.ncbi.nlm.nih.gov/clinvar/) or other comparative means. An additional patient was included as concordant (mapped to REARR) as their pathogenic t*BRCA1* CNA was deemed to align with a g*BRCA1* deletion of exons 13–15. Of the three non-concordant patients, one patient exhibited a g*BRCA2* SNV that was not detected in the tumor, which exhibited a different *BRCA2* SNV of unknown pathogenicity; the second patient exhibited a g*BRCA2* duplication of exons 15–18, which was not detected in the tumor, and a t*BRCA2* SNV of unknown pathogenicity (this patient was mapped to REARR category); and the third patient exhibited a g*BRCA1* rearrangement (del exon 16), which was not detected in the tumor, and a t*BRCA1* splice site mutation (this patient was mapped to REARR category). *BRCA1/2* *breast cancer susceptibility gene 1 or 2*, DEL deletion, g*BRCA1/2*mut germline *BRCA1/2* mutation, INS insertion, REARR rearrangement, SNV single nucleotide variant, t*BRCA* tumor *BRCA*.

Concordance between g*BRCA1/2* and t*BRCA1/2* mutational status was evaluated in 56 patients in the ITT population who were analyzed using the BRACAnalysis CDx^®^ assay and had tumor tissue evaluable using FoundationOne^®^ CDx. Here, 53 patients (95%) exhibited concordance in mutations, i.e., same mutation detected in germline also found in tumor, and 54 patients (96%) exhibited concordance in mutational status, i.e., same *BRCA* gene mutated in germline also mutated in tumor (Fig. 2).

*BRCA* LOH, with retention of a mutant *BRCA* allele, was predicted in 40/47 (85%) t*BRCA1/2*mut patients evaluable for *BRCA* zygosity (Table 2, Fig. 1). Of these 40 patients, 37 exhibited tumor retention of a known g*BRCA*mut. Of the remaining three of 40 patients, one (patient 60) exhibited *BRCA* LOH with tumor retention of a presumed somatic (i.e., not detected in germline testing) *BRCA1* mutation, with a different known g*BRCA*mut predicted to be in a heterozygous state in the tumor; g*BRCA*mut details were not available for the other two patients (patients 21 and 37) (Supplementary Table 1).

### Prevalence of non-*BRCA1/2* tumor mutations

*TP53* and *PIK3CA* were the most prevalent non-*BRCA* tumor mutations. In both cohorts, *TP53* mutations were more prevalent with *BRCA1*mut than *BRCA2*mut; this trend was particularly evident in Cohort 1 (comprising patients with a prior platinum response; Table 3). In both cohorts, *PIK3CA* mutations were more prevalent in *BRCA2*mut tumors versus tumors harboring *BRCA1*mut (Table 3), with differences in mutation incidence reflecting tumor subtype differences: 5/6 patients with *PIK3CA* mutations had t*BRCA2*mut hormone-receptor positive (HR+) disease, while the remaining patient had t*BRCA1*mut triple-negative breast cancer (TNBC) (data not shown).

**Table 3:** Most prevalently mutated non-*BRCA1/2* genes in *BRCA1/2*-mutated patients (evaluable ITT population)^a^.

Gene mutations	Cohort 1 (*n* = 29)	Cohort 2 (*n* = 28)	Total Cohorts 1 and 2 (*N* = 57)
Copy number alterations excluded (%)			
*TP53*			
* BRCA1*	88.2	58.3	75.9
* BRCA2*	8.3	18.8	14.3
* BRCA1/2*	55.2	35.7	45.6
*PIK3CA*			
* BRCA1*	5.9	0.0	3.4
* BRCA2*	16.7	18.8	17.9
* BRCA1/2*	10.3	10.7	10.5
Copy number alterations only (%)			
*RAD21*			
* BRCA1*	17.6	41.7	27.6
* BRCA2*	25.0	43.8	35.7
* BRCA1/2*	20.7	42.9	31.6
*MYC*			
* BRCA1*	11.8	33.3	20.7
* BRCA2*	8.3	12.5	10.7
* BRCA1/2*	10.3	21.4	15.8

Cohort 1 comprised patients with response to prior platinum and no progression within 8 weeks and Cohort 2 comprised patients who received ≥3 platinum-free cytotoxic regimens.

*BRCA1/2* breast cancer susceptibility gene 1 or 2, *ITT* intent-to-treat.

^a^Evaluable ITT population includes all patients with tumor samples suitable for the genomic evaluation and analyzed using FoundationOne^®^ CDx who have *BRCA1/2* mutations (known or likely pathogenic impact, excluding copy number alterations). Genes shown are mutated in ≥10% of patients in combined cohorts.

When analysis was confined to CNAs in the ABRAZO population, *RAD21* and *MYC* were the most frequently altered non-*BRCA* genes in *BRCA*-mutated tumors (only amplification events detected; see Fig. 1). Furthermore, CNAs of *RAD21* and *MYC* were more commonly observed in tumors from Cohort 2 than in Cohort 1 (Table 3).

### Genomic LOH

In the evaluable ITT population, the median (range) gLOH score was 21.3% (9.1–41.8) and 23.4% (0.0–38.9) for Cohorts 1 and 2, respectively. Across both cohorts, 81.6% (31/38) of patients had gLOH ≥16% (exploratory threshold for high gLOH)^20^, with similar results observed in Cohort 1 (85.0% [17/20 patients]) and Cohort 2 (77.8% [14/18 patients]) separately. Of the seven evaluable patients with gLOH <16% (patients 8, 34, 49, 51, 55, 66, and 71), five had *BRCA* LOH, one did not exhibit *BRCA* LOH, and one was not evaluable for *BRCA* LOH (Supplementary Table 1).

Of 34 patients from combined Cohorts 1 and 2 who were evaluable for both gLOH and *BRCA* LOH status, only three lacked *BRCA* LOH (Supplementary Table 1 [patients 51, 60, 67]), precluding assessment of the relationship between gLOH and *BRCA* LOH in this study.

### Clinical benefit and tumor mutational profile

In Cohort 1, the clinical benefit rate at 24 weeks (CBR24) was 24% (4/17; 95% CI 7–52) and 25% (3/12; 95% CI 5–57) for t*BRCA1*mut and t*BRCA2*mut, respectively. In Cohort 2, the CBR24 was 67% (8/12; 95% CI 35–90) and 63% (10/16; 95% CI 35–85) for t*BRCA1*mut and t*BRCA2*mut, respectively. A range of clinical outcomes were reported in t*BRCA*mut patients lacking *BRCA* LOH (*n* = 2 in Cohort 1; *n* = 5 in Cohort 2); although only two patients achieved a partial response, three had stable disease, and PFS ranged from 1.35–30.29 months (Supplementary Table 1). The low number of t*BRCA*mut patients without *BRCA* LOH (*n* = 7) precluded efficacy comparisons between t*BRCA*mut patients exhibiting or not exhibiting *BRCA* LOH.

**Fig. 3:**
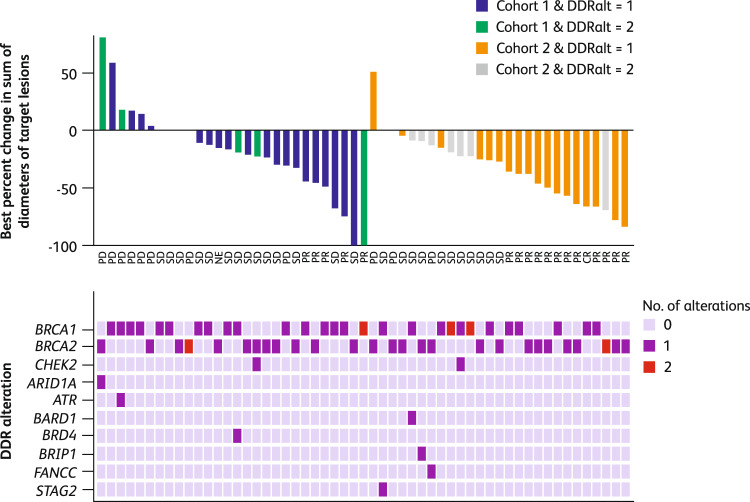
Best percent change of sum of diameters of target lesions from baseline over time by investigator assessment – by number of DDR alterations^1^. ^1^Based on evaluable ITT population with measurable disease. Number of DDR alterations is sum of known and likely pathogenic variants in the following genes, excluding copy number alterations: *BRCA1, BRCA2, CHEK2, ARID1A, ATR, BARD1, BRD4, BRIP1, FANCC, STAG2*. *BRCA1/2* *breast cancer susceptibility gene 1 or 2*, CR complete response, DDR DNA damage response, DDRalt DNA damage response alteration, ITT intent-to-treat, NE non-evaluable, PD progressive disease, PR partial response, SD stable disease.

A significant association was observed between the number of DDR alterations (two vs one) and best response to talazoparib in Cohort 2, with single mutations being associated with higher responsiveness (Fig. 3; odds ratio [OR] 0.08, 95% CI 0.01–0.83, *p* = 0.03). However, analysis of Cohort 1 did not show such an association (Fig. 3; OR 0.85, 95% CI 0.08–9.44, *p* = 1). In addition, there was no significant association between the number of DDR alterations (two vs one) and CBR24 in Cohort 1 or 2 (OR 1.7, 95% CI 0.24–12.17, *p* = 0.62, and OR 0.36, 95% CI 0.06–2.00, *p* = 0.37, respectively). The presence of non-*BRCA* DDR mutations did not appear to enhance talazoparib sensitivity in this *BRCA*-mutant setting (Fig. 3).

In the analysis exploring the impact of common non-DDR alterations on PFS, no associations were evident between the alteration status of *TP53* or *RAD21*, and PFS in Cohort 1 or 2 (Table 4).

**Table 4:** Progression-free survival according to alteration status of selected non-DDR genes (t*BRCA*mut ITT population).

Evaluable ITT population with tumors bearing *BRCA1/2* mutations	Cohort 1 (*n* = 29)		Cohort 2 (*n* = 28)	
	Mutations	CNAs	Mutations	CNAs
*TP53* mutation				
HR (95% CI)	1.453 (0.682 to 3.096)	NE	0.612 (0.273 to 1.373)	NE
* n*, altered/unaltered	16/13		10/18	
* n*, events altered/unaltered	16/13		9/18	
* p* value	0.3254	NE	0.2269	NE
*RAD21* amplification				
HR (95% CI)	NE	NE	NE	0.815 (0.378 to 1.756)
* n*, altered/unaltered				12/16
* n*, events altered/unaltered				12/15
* p* value	NE	NE	NE	0.5984

Cohort 1 comprised patients with response to prior platinum and no progression within 8 weeks and Cohort 2 comprised patients who received ≥3 platinum-free cytotoxic regimens.

Only select subgroup comparisons are displayed for *TP53* and *RAD21* (those where both subgroups had ≥10 patients), otherwise analyses deemed NE. *MYC*, *PTEN*, and *PIK3CA* are not displayed since both mutant/CNA (i.e., alteration) and non-mutant/non-CNA (i.e., unaltered) subgroups had <10 patients.

Cox proportional hazards model with unaltered as the reference group was used to calculate HR and 95% CI. HR < 1 indicates better PFS in altered group, while HR > 1 indicates better PFS in the unaltered group. Log-rank two-sided test was performed to compare between altered/unaltered groups. *BRCA* mutations are defined as known or likely pathogenic *BRCA* variants (*BRCA* CNAs excluded). For *TP53* and *RAD21*, mutations are defined as known or likely pathogenic variants (CNAs excluded), with known or likely pathogenic CNAs displayed separately^19^.

*BRCA1/2* *breast cancer susceptibility gene 1* or *2*, *CI* confidence interval, *CNA* copy number alteration, *DDR* DNA damage response, *HR* hazard ratio, *ITT* intent-to-treat, *NE* non-evaluable, *PFS* progression-free survival, tBRCAmut tumor *BRCA* mutation.

## Discussion

In these analyses of tumor tissue from patients enrolled in the open-label, Phase 2 ABRAZO study, 97% of evaluable tumors exhibited ≥1 *BRCA1/2*mut and there was 95% concordance between known g*BRCA1/2*mut and t*BRCA1/2*mut; this is perhaps unsurprising given the importance of g*BRCA*mut in breast cancer pathology, and the fact that patients were selected based on g*BRCA*mut status.

*BRCA* LOH was evident in 85% of t*BRCA*mut patients evaluable for *BRCA* zygosity. This high prevalence of LOH for *BRCA1/2*mut is consistent with previous studies in breast cancer where loss of the wildtype chromosome was seen in 88–89% of *BRCA1/2*mut patients^18,21^. Sequencing of another set of g*BRCA1/2*mut breast tumors also showed high incidence of locus-specific LOH for *BRCA1* (90%); however, lower LOH incidence (54%) was observed for *BRCA2*. In that dataset, LOH for *BRCA1* was more commonly copy neutral and loss of the wildtype allele more frequent in g*BRCA2*mut tumors^16^. In a larger patient cohort containing pan-cancer germline pathogenic *BRCA1/2* carriers, 86% of zygosity changes targeted loss of the remaining wildtype allele^22^. This is consistent with a positive selective pressure for bi-allelic inactivation of *BRCA1/2*^22^. Of note, there are mechanisms of silencing the wildtype *BRCA* allele other than *BRCA* LOH, such as *BRCA1* promoter methylation; hence, absence of *BRCA* LOH does not necessarily correspond to partial retention of wildtype *BRCA* function^16,21,23^. Studies have also suggested that a haploinsufficiency phenotype in g*BRCA2*mut cells results in reduced functional BRCA2 protein levels, which could contribute toward chromosomal instability and subsequent promotion of tumorogenesis^24,25^.

*BRCA1/2* alterations are most frequently bi-allelic in tumor types that have demonstrated clinical sensitivity to PARPi monotherapy, including ovarian, breast, prostate, and pancreatic cancer^18,22^. Bi-allelic *BRCA1/2* inactivating mutations are also associated with Signature 3, a pattern of genome-wide mutations linked to HRD in breast cancer^23^. However, the low fraction of tumors without *BRCA* LOH in this study precluded the assessment of impact of zygosity on outcome.

DDR gene alteration burden or alteration status of selected non-*BRCA* genes was not generally associated with clinical efficacy in this study, as assessed by best percent change of sum of longest diameters of target lesions from baseline over time, or PFS, respectively. Moreover, the presence of additional non-*BRCA* DDR mutations was not associated with enhanced talazoparib efficacy. Tumor HRD (as assessed by gLOH) was variable, but high, in ABRAZO. However, low patient numbers precluded correlations with efficacy.

Previously, gLOH has been used to determine deficiency in homologous recombination in tumor samples^26^ and higher scores have been associated with better therapeutic response^26,27^. gLOH scores were on average relatively high in ABRAZO and similar to those found in HER2– g*BRCA1/2*mut breast cancer (median 23.0%, based on *N* = 1730 tumors; 27.8% for g*BRCA1*mut and 21.0% for g*BRCA2*mut) from Foundation Medicine’s FoundationCore^®^ database. Moreover, these scores are much greater than those seen for the overall breast cancer population (median 12.2%, based on *N* = 20,614 tumors), reflecting HRR deficiency associated with g*BRCA1/2*mut. In addition, ABRAZO patients exhibited a relatively high observed fraction of gLOH-high tumors (≥16% gLOH score^20^), which was also similar to that reported in HER2– g*BRCA1/2*mut breast cancer (78.1%; 82.3% for g*BRCA1*mut and 74.9% for g*BRCA2*mut) and over two-fold higher than that observed in the overall breast cancer population (35.3%) in the Foundation Medicine database. The association of g*BRCA*mut status with elevated gLOH was also evident within both HER2– and TNBC disease subtypes in the Foundation Medicine database (Supplementary Fig. 2).

Breast tumors often display distinct mutational profiles and gene rearrangement signatures that are associated with *BRCA*mut^21^. *TP53* and *PIK3CA* are among the most frequently mutated genes in HR+/HER2− breast cancer^28^. In the Foundation Medicine database, *TP53* mutations were evident in 86.2% (225/261) and 30.1% (96/319) of g*BRCA1*mut and g*BRCA2*mut tumors, respectively (Q = 1.38E–44), after Benjamini-Hochberg correction for multiple comparisons. In another dataset of pan-disease *BRCA1/2*-mutated cancers, *TP53* mutations were the most common genomic alterations overall (67%) and were most prevalent in g*BRCA1*mut carriers^29^. Furthermore, breast and ovarian tumors with g*BRCA1/2*mut are more likely to have *TP53* mutations if they display *BRCA* LOH^16^. The strong correlation between *BRCA* mutations and *TP53* mutations reflects a common association with TNBC^30,31^. Similarly, in the ABRAZO population, *TP53* mutations were more prevalent in *BRCA1*mut than *BRCA2*mut tumors. Somatic loss of both BRCA1 and TP53 has been recapitulated in animal models and results in rapid formation of highly proliferative, poorly differentiated, estrogen receptor-negative mammary carcinomas^32^, suggesting a role for *TP53* mutations in this setting. Furthermore, studies have shown that p53 interacts with *BRCA1* and regulates the ability of *BRCA1* to respond to DNA damage, suggesting that wildtype *BRCA1* can be rendered dysfunctional in a mutated *TP53* background^33,34^.

In the Foundation Medicine database, *PIK3CA* mutations were evident in 8.4% (22/261) and 13.2% (42/319) of g*BRCA1*mut and g*BRCA2*mut tumors, respectively (Q = 0.08). Similarly, in the ABRAZO population, a numerically higher prevalence of *PIK3CA* mutations was associated with *BRCA2*mut tumors, particularly *BRCA2*mut HR+ tumors. These findings reflect previous studies which demonstrate that *PIK3CA* mutations are frequently found in HR+/HER2− breast cancer^35^. In a group of patients with hereditary breast cancer, *PIK3CA* mutations were associated with *BRCA2* but not *BRCA1* mutations, and with luminal-type breast cancer^36^.

Here, several other non-*BRCA* gene mutations were detected in tumors including *CHEK2*, *ARID1A*, *ATR*, *BARD1*, *BRD4*, *BRIP1*, *FANCC*, and *STAG2*. Mutations in *ARID1A*, a subunit of the SWI/SNF chromatin remodeling complex, represent the most frequent alteration of the SWI/SNF complex in estrogen receptor-positive breast cancer, and *ARID1A* has been suggested to play a major role in breast luminal lineage fidelity and endocrine therapy sensitivity^37^.

The clinical benefit of talazoparib in the ABRAZO population was comparable between cohorts for patients with *BRCA1*mut or *BRCA2*mut tumors. Despite only representing ~15% of evaluable patients in the ABRAZO population, there was also potential for clinical benefit of talazoparib in t*BRCA1/2*mut patients lacking *BRCA* LOH. DDR deficiencies elicited by mutations, for example, in *BRCA1/2*, are associated with a high mutational burden or genomic instability with worse clinical outcomes across almost all cancer types^38^. Here, a significant association was observed between the number of DDR alterations and best response to talazoparib in Cohort 2. However, there was no significant association between the number of DDR gene alterations and CBR24. Of note, the presence of non-*BRCA1/2* DDR mutations did not appear to enhance sensitivity to talazoparib in patients with *BRCA1/2*mut; this finding was expected given that patients were enrolled based on g*BRCA*mut status and the importance of g*BRCA*mut in tumor pathobiology in such patients, potentially suggesting that the observation in Cohort 2 was a chance finding. Furthermore, no associations were evident between the alteration status of *TP53* and *RAD21*, and PFS in Cohorts 1 or 2.

Limitations of the ABRAZO study have previously been discussed and include the termination of enrollment prior to completion, resulting in a low number of evaluable patients in each cohort^12^. This was due to overlapping enrollment criteria with the Phase 3 EMBRACA trial (NCT01945775)^39^ following a protocol amendment to EMBRACA^12^. Early termination also precluded further stratification by *BRCA1/2*mut and breast cancer subtypes. Furthermore, DNA sequencing may fail to find functional non-genetic deficiencies in DDR genes (e.g., promoter methylation). Finally, the primary/metastatic origin of archival tissue was not determined for this study. To address some of these limitations, similar analyses have been performed for tumor tissue from the Phase 3 EMBRACA study^40^. Whole genome sequencing/next-generation sequencing (NGS) analyses of paired biopsies from ABRAZO and EMBRACA are also pending to address acquired resistance mechanisms.

In this genomic analysis of the ABRAZO trial, we demonstrate that tumor-only *BRCA1/2* sequencing has high sensitivity for g*BRCA1/2*mut. We report the genomic profile of *BRCA1/2*-related breast cancer, and provide evidence that non-*BRCA* genetic/genomic events did not appear to impact the efficacy of talazoparib. These findings are consistent with those recently published for the Phase 3 EMBRACA (talazoparib) and OlympiAD (olaparib) studies^40,41^. As both germline and somatic mutations may be identified by tumor sequencing, further research is required to assess whether tumor-only sequencing can direct talazoparib therapy.

## Methods

### Study design and patients

ABRAZO was an open-label, two-cohort, Phase 2 study of talazoparib (1 mg, orally once daily) in patients with MBC with a deleterious or a suspected deleterious g*BRCA1/2*mut^12^. Briefly, the study comprised two cohorts: Cohort 1 included patients who had a complete response or partial response to a previous platinum-containing regimen for metastatic disease, and no disease progression within 8 weeks of the last dose of platinum therapy; Cohort 2 included patients who had received ≥3 previous cytotoxic chemotherapy regimens for metastatic disease and no previous platinum therapy for metastatic disease. Patients with HER2-positive disease were eligible for either cohort, provided they were considered refractory to HER2-targeted therapy^12^. The primary and secondary endpoints were ORR and CBR24, respectively. The protocol was approved by the appropriate Institutional Review Board or local ethics committee at each participating institution and written informed patient consent was obtained^12^. The following independent ethics committees or Institutional Review Boards provided study approval: Comité de Protection des Personnes Sud-Ouest et Outre Mer III, Bordeaux, France; Ethik-Kommission der Medi, Fakultät der Ludwig-Maximilians- Universität (LMU) München – Fachbereich Medizin, München, Germany; Comité Éticos de Investigación Clínica, Hospital Universitario Ramón y Cajal, Madrid, Spain; NRES Committee London - City and East, Bristol Research Ethics Committee Centre, Bristol, UK; Office of the Human Research Protection Program, Los Angeles, CA, USA; Johns Hopkins Medicine Institutional Review Board, Baltimore, MD, USA; The Committee on Human Research, University of California, San Francisco, CA, USA; Western Institutional Review Board, Puyallup, WA, USA; Penn State College of Medicine Institutional Review Board, Hershey, PA, USA; University of Texas MD Anderson Cancer Center Institutional Review Board, Houston, TX, USA; University of Miami Institutional Review Board, Miami, FL, USA; University of Tennessee Graduate School of Medicine Institutional Review Board, Knoxville, TN, USA; Spectrum Health Institutional Review Board, Grand Rapids, MI, USA; Administrative Panels on Human Subjects in Medical Research, Stanford University, Palo Alto, CA, USA; and the Memorial Sloan Kettering Cancer Center Institutional Review Board, New York, NY, USA. The ethics committees were properly constituted and compliant with all requirements and local regulations. The study was conducted in accordance with the protocol, good clinical practice standards, the Declaration of Helsinki, and the International Conference on Harmonization.

### Next-generation sequencing and mutational analysis

In the majority of patients, g*BRCA1/2*mut were determined using the BRACAnalysis CDx^®^ assay (Myriad Genetics Inc., Salt Lake City, UT, USA). Enrollment of five patients was supported by local *BRCA1/2* testing^12^. Archival or de novo tumor tissue (formalin-fixed, paraffin-embedded tissue; primary/metastatic sites) was sequenced using the FoundationOne^®^ CDx NGS panel (Foundation Medicine, Inc., Cambridge, MA, USA), including mutations in *BRCA1/2* and non-*BRCA* genes involved in DDR. For the purposes of this analysis, tumor mutations were defined as known or likely pathogenic variants per the FoundationOne^®^ CDx test with CNAs excluded.

The influence of tumor *BRCA1/2* mutational zygosity on PFS was explored by comparing patients with and without *BRCA1/2* LOH. gLOH and somatic-germline-zygosity (SGZ) assessments were performed by Foundation Medicine Inc. using the Foundation Core Build 2019Q1^42,43^.

DNA was extracted and adaptor ligated hybridization capture for all coding exons of 310 genes plus 34 introns frequently rearranged in cancer was performed. Libraries were sequenced to a median unique coverage depth of >500X. Analysis for genomic alterations, including short variant alterations (base substitutions, insertions, and deletions), copy number alterations (amplifications and homozygous deletions), as well as gene rearrangements was performed as previously described^44^.

To assess tumor and germline concordance, mutations were mapped to a common Variation ID in ClinVar (https://www.ncbi.nlm.nih.gov/clinvar/) or other comparative means. In addition, non-*BRCA* DDR genes (*CHEK2*, *ARID1A*, *ATR*, *BARD1*, *BRD4*, *BRIP1*, *FANCC*, *STAG2*) were selected for inclusion in correlative analyses on the basis of involvement in homologous recombination-mediated DNA repair and/or demonstrated potential for mutations to sensitize to PARP inhibitors in nonclinical models^45–48^, coupled with presence of known or likely pathogenic variants (excluding CNAs) of these genes in this dataset.

### Foundation Medicine clinical database

The Foundation Medicine clinical database comprises patient cases that underwent genomic profiling as a routine part of clinical care using a targeted comprehensive genomic profiling assay in a Clinical Laboratory Improvement Amendments (CLIA)-certified, College of American Pathologists (CAP)-accredited, New York State-approved laboratory (FoundationOne^®^ CDx, Cambridge, MA, USA). Database version Foundation Core Build 2019Q1 was used in this study.

### Endpoint definitions in ABRAZO

ORR was defined as the proportion of patients in the tumor-evaluable population who had a confirmed objective response (best overall response of complete or partial response) assessed by the independent radiology facility using Response Evaluation Criteria In Solid Tumors (RECIST) version 1.1 at the time of data cutoff. CBR24 was defined as complete response, partial response, or stable disease ≥24 weeks per RECIST version 1.1 by investigator assessment.

### Statistical analysis

The influence of tumor *BRCA1/2* mutational zygosity on PFS was analyzed by comparison of patients with and without *BRCA1/2* LOH using the Cox proportional hazards model and a log-rank two-sided test to compare between altered/unaltered groups. Logistic regression was used to determine the odds ratio, 95% CI, and *p* value for the effect of two versus one DDR mutations on PFS.

The Mann–Whitney U test was used for comparison of gLOH values between germline BRCA wildtype and germline BRCA-mutated tumors in patients with HER2– and TNBC and Fisher’s exact test was used to determine the odds ratio and *p* value for comparison of the percentage of samples with gLOH ≥16% between the two groups. No corrections were made for multiple comparisons due to the low patient numbers and exploratory nature of this research, and as this study is primarily intended for hypothesis-generation.

### Reporting summary

Further information on research design is available in the Nature Research Reporting Summary linked to this article.

### Supplementary information

Supplemental Material

Reporting Summary

## Data Availability

This study presents a secondary analysis of data from the ABRAZO trial^12^ and Pfizer does not have access to the primary sequencing files. Upon request, and subject to review, Pfizer will provide the clinical data that support the findings of this study. Subject to certain criteria, conditions and exceptions, Pfizer may also provide access to the related individual anonymized participant data. See https://www.pfizer.com/science/clinical-trials/trial-data-and-results for more information.
