# Supplementary material for: Genomic analysis of advanced breast cancer tumors from talazoparib-treated gBRCA1/2mut carriers in the ABRAZO study
Source: NPJ Breast Cancer. 2023 Oct 6;9:81. doi: 10.1038/s41523-023-00561-y (PMC10558443; doi:10.1038/s41523-023-00561-y)
Supplement: Supplementary file 1 — Supplemental Material [file 41523_2023_561_MOESM1_ESM.pdf]

Supplementary Materials

Supplementary Table 1. *BRCA* variant profile and selected clinical outcomes (evaluable ITT population)

				Tumor			Tumor SGZ prediction					
Patient/ cohort	g*BRCA1* mutation	g*BRCA2* mutation	g*BRCA* mutation details	*BRCA1*/ *BRCA2*	Pathogenic status	Mutation (protein)	Somatic/ germline	Zygosity	Tumor gLOH (%)	Clinical benefit[1]	Best response[2]	PFS (months)[3]
1/ 1	N/A	Y	2157delG							Y	SD	8.28
2/ 1	Y	N/A	187delAG							N	SD	2.99
3/ 1	Y	N/A	4370delGT							N	PD	1.41
4/ 1	N	N		*BRCA1*	Known	211A>G (R71G)	Germline	NE	NE	N	SD	4.67
5/ 1	Y	N/A	153C>T (Q12X)	*BRCA1*	Likely	34C>T (Q12*)	Germline	Homozygous	21.61	Y	PR	5.29
6/ 1	N/A	Y	8396A>G (D2723G)	*BRCA2*	Known	8168A>G (D2723G)	Germline	NE	NE	N	SD	7.13
7/ 1	N/A	Y	IVS17-1G>C	*BRCA2*	Likely	7977-1G>C (splice site 7977-1G>C)	NE	Homozygous	16.06	Y	PR	16.79
8/ 1	N/A	Y	8475delGA	*BRCA2*	Likely	8247_8248delGA (K2750fs*13)	Germline	Homozygous	11.61	N	SD	5.32
9/ 1	Y	N/A	1569G>T (G484X)	*BRCA1*	Likely	1450G>T (G484*)	Germline	Homozygous	30.73	N	SD	3.71
10/ 1	Y	N/A	187delAG							N	SD	4.63
11/ 1	Y	N/A	5622C>T (R1835X)	*BRCA1*	Known	5503C>T (R1835*)	Germline	Homozygous	27.68	N	SD	2.76
				BRCA2	Unknown	695A>G (Y232C)	Germline	Heterozygous				

				Tumor			Tumor SGZ prediction					
Patient/ cohort	g*BRCA1* mutation	g*BRCA2* mutation	g*BRCA* mutation details	*BRCA1*/ *BRCA2*	Pathogenic status	Mutation (protein)	Somatic/ germline	Zygosity	Tumor gLOH (%)	Clinical benefit[1]	Best response[2]	PFS (months)[3]
12/ 1	N	N		*BRCA2*	Likely	5238_5239insT (N1747fs*1)	NE	Heterozygous	NE	N	PD	1.35
				BRCA2	Likely	5191_5219>GAAAAAA (H1731fs*39)	Subclonal Somatic	NE				
13/ 1	Y	N/A	2576delC	*BRCA1*	Unknown	4910C>T (P1637L)	Germline	NE	23.29	N	SD	3.12
				BRCA1	Likely	2457delC (D821fs*25)	NE	NE				
14/ 1	Y	N/A	4184del4	*BRCA1*	Likely	4065_4068delTCAA (N1355fs*10)	NE	NE	20.46			1.22
15/ 1	N/A	Y	8395G>C (D2723H)	*BRCA2*	Known	8167G>C (D2723H)	Germline	Homozygous	35.1	Y	SD	8.31
16/ 1	Y	N/A	del exons 13-15	*BRCA1*	Known	Copy Number Alteration	NE	NE	NE	Y	CR	41.43
17/ 1	Y	N/A	5385insC	*BRCA1*	Likely	5266_5267insC (Q1756fs*74)	Germline	NE	NE	N	PD	2.50
18/ 1	Y	N/A	3875del4	*BRCA1*	Likely	3756_3759delGTCT (S1253fs*10)	NE	Homozygous	NE	N	SD	10.22
				BRCA2	Unknown	9976A>T (K3326*)	Germline	Heterozygous				
19/ 1	N/A	Y	1534delAA							N	SD	2.69
20/ 1	N/A	Y	8395G>C (D2723H)							Y	PR	19.35
21/ 1	N	N		*BRCA1*	Likely	68_69delAG (E23fs*17)	Germline	Homozygous	NE	N	SD	5.55
22/ 1	N/A	Y	6265A>T (K2013X)	*BRCA2*	Likely	6037A>T (K2013*)	Germline	Homozygous	17.4	N	NE	2.14
23/ 1	Y	N/A	300T>G (C61G)	*BRCA1*	Known	181T>G (C61G)	Germline	Homozygous	39.49	Y	PR	5.55
24/ 1	N/A	Y	5466insT							N	PD	1.41

Patient/ cohort	g*BRCA1* mutation	g*BRCA2* mutation	g*BRCA* mutation details	Tumor *BRCA1*/ *BRCA2*	Pathogenic status	Mutation (protein)	Tumor SGZ prediction Somatic/ germline	Zygosity	Tumor gLOH (%)	Clinical benefit[1]	Best response[2]	PFS (months)[3]
25/ 1	Y	N/A	IVS5-12A>G							Y	CR	7.62
26/ 1	Y	N/A	399C>T (Q94X)							N	PD	1.38
27/ 1	N/A	Y	2665del8							Y	SD	9.69
28/ 1	N/A	Y	9325insA							Y	PR	8.41
29/ 1	Y	N/A	5385insC							Y	SD	8.28
30/ 1	N/A	Y	9345G>C (P3039P)	*BRCA2*	Unknown	5070A>C (K1690N)	Germline	NE	NE	Y	PR	8.54
31/ 1	N	N								Y	PR	5.55
32/ 1	N/A	Y	4075delGT	*BRCA2*	Likely	3847_3848delGT (V1283fs*2)	Germline	Homozygous	17.03	N	PD	2.37
33/ 1	N/A	Y	635delA							N	PD	2.30
34/ 1	N/A	Y	3064delGA	*BRCA2*	Likely	2836_2837delGA (D946fs*12)	Germline	Homozygous	9.1	N	SD	4.01
35/ 1	Y	N/A	IVS5+2T>C	*BRCA1*	Likely	Rearrangement (BRCA1-TPM2)	NE	NE	21.05	Y	PR	15.44
				BRCA1	Likely	212+2T>C (splice site 212+2T>C)	Germline	Homozygous				
36/ 1	N/A	Y	dup exons 15-18	*BRCA2*	Unknown	7052C>T (A2351V)	NE	NE	23.14	N	PD	1.18
37/ 1	N	N		*BRCA1*	Likely	3598C>T (Q1200*)	Germline	Homozygous	41.79	N	PD	1.41
38/ 1	Y	N/A	962del4							N	SD	3.94

Patient/ cohort	g*BRCA1* mutation	g*BRCA2* mutation	g*BRCA* mutation details	Tumor *BRCA1*/ *BRCA2*	Pathogenic status	Mutation (protein)	Tumor SGZ prediction Somatic/ germline	Zygosity	Tumor gLOH (%)	Clinical benefit[1]	Best response[2]	PFS (months)[3]
39/ 1	N/A	Y	9174insA	*BRCA2*	Unknown	978C>A (S326R)	Germline	Homozygous	NE	N	PD	1.48
				BRCA2	Likely	8946_8947insA (D2983fs*35)	NE	Homozygous				
40/ 1	N/A	Y	5910C>G (Y1894X)	*BRCA2*	Likely	5682C>G (Y1894*)	Germline	Homozygous	NE	N	SD	3.12
41/ 1	Y	N/A	3604delA	*BRCA1*	Likely	3485delA (D1162fs*48)	Germline	Homozygous	18.69	N	PD	1.35
42/ 1	N/A	Y	5277insT	*BRCA2*	Likely	5049_5050insT (T1684fs*5)	Germline	Heterozygous	NE	Y	SD	8.28
43/ 1	N/A	Y	3386T>G (L1053X)	*BRCA2*	Likely	3158T>G (L1053*)	Germline	Homozygous	20.16	N	SD	3.94
44/ 1	Y	N	IVS16+3G>C	*BRCA1*	Known	4986+3G>C (splice site 4986+3G>C)	NE	NE	NE	N	SD	2.63
				BRCA2	Unknown	6523G>C (E2175Q)	NE	Heterozygous				
45/ 1	Y	N/A	330A>G (R71G)	*BRCA1*	Known	211A>G (R71G)	Germline	Homozygous	33.59	N	PD	1.15
46/ 1	Y	N/A	4400ins39							Y	PR	5.36
47/ 1	N/A	Y	8395G>C (D2723H)							Y	SD	7.69
48/ 1	Y	N	5622C>T (R1835X)	*BRCA1*	Known	5503C>T (R1835*)	Germline	Homozygous	26.49	Y	PR	4.21
				BRCA2	Unknown	1385A>G (E462G)	Germline	Heterozygous				
49/ 1	Y	N/A	943ins10	*BRCA1*	Likely	824_825insAGCCATGTGG (T276fs*14)	Somatic	Homozygous	9.7	N	PD	1.48
50/ 2	N/A	Y	5638delGT	*BRCA2*	Likely	5410_5411delGT (V1804fs*2)	Germline	Heterozygous	NE	Y	PR	5.49

Patient/ cohort	g*BRCA1* mutation	g*BRCA2* mutation	g*BRCA* mutation details	Tumor *BRCA1*/ *BRCA2*	Pathogenic status	Mutation (protein)	Tumor SGZ prediction Somatic/ germline	Zygosity	Tumor gLOH (%)	Clinical benefit[1]	Best response[2]	PFS (months)[3]
51/ 2	N/A	Y	2041delA	*BRCA1*	Unknown	199G>T (D67Y)	Germline	NE	15.48	Y	PR	5.45
				BRCA2	Likely	1826_1841delAAAAATCAGAACTAAT (Q609fs*30)	Somatic	Heterozygous				
				BRCA2	Likely	1813delA (I605fs*9)	NE	NE				
				BRCA2	Unknown	10127C>T (S3376L)	Somatic	Heterozygous				
52/ 2	Y	N/A	4154delA							N	PD	2.83
53/ 2	N/A	Y	6092C>A (S1955X)	*BRCA2*	Likely	5864C>A (S1955*)	Germline	NE	NE	Y	PR	7.20
54/ 2	N/A	Y	9254del5	*BRCA2*	Likely	9026_9030delATCAT (Y3009fs*7)	Germline	Homozygous	16.03	Y	PR	15.64
55/ 2	Y	N/A	3596del4	*BRCA1*	Likely	3477_3480delAAAG (I1159fs*50)	NE	Homozygous	14.43	Y	PR	9.00
				BRCA2	Unknown	Rearrangement (BRCA2-PDS5B)	NE	NE				
56/ 2	Y	N/A	del exon 16	*BRCA1*	Likely	4484+1G>A (splice site 4484+1G>A)	NE	Homozygous	NE	N	SD	5.68
57/ 2	Y	N/A	1459insG	*BRCA1*	Likely	1340_1341insG (H448fs*8)	NE	Homozygous	NE	Y	PR	5.45
58/ 2	N/A	Y	6027del4	*BRCA2*	Likely	5799_5802delCCAA (N1933fs*29)	Germline	Homozygous	NE	Y	PR	5.55
59/ 2	Y	N/A	5385insC							Y	PR	8.25
60/ 2	N/A	Y	F1870X	*BRCA1*	Unknown	4318G>C (E1440Q)	NE	Homozygous	27.28	N	PD	1.41
				BRCA2	Likely	5609_5610TC>AG (F1870*)	Germline	Heterozygous				
61/ 2	Y	N/A	IVS18-1G>A	*BRCA1*	Likely	Rearrangement (BRCA1-CIITA)	NE	NE	23.28	N	SD	4.21
				BRCA1	Likely	5153-1G>A (splice site 5153-1G>A)	Germline	Homozygous				
62/ 2	N/A	Y	5897del5	*BRCA2*	Likely	5669_5673delTGGCA (M1890fs*8)	Germline	Homozygous	38.93	Y	SD	7.75

				Tumor			Tumor SGZ prediction					
Patient/ cohort	g*BRCA1* mutation	g*BRCA2* mutation	g*BRCA* mutation details	*BRCA1*/ *BRCA2*	Pathogenic status	Mutation (protein)	Somatic/ germline	Zygosity	Tumor gLOH (%)	Clinical benefit[1]	Best response[2]	PFS (months)[3]
63/ 2	N/A	Y	2683C>T (Q819X)							Y	PR	5.62
64/ 2	N/A	Y	6079del4	*BRCA2*	Likely	5851_5854delAGTT (S1951fs*11)	NE	Homozygous	NE	N	SD	4.11
65/ 2	N/A	Y	3058A>T (K944X)							Y	PR	4.44
66/ 2	Y	N	187delAG	*BRCA1*	Likely	68_69delAG (E23fs*17)	NE	Homozygous	0.01	Y	CR	33.15
67/ 2	Y	N/A	3726C>T (R1203X)	*BRCA1*	Likely	61_62insA (I21fs*20)	Somatic	Heterozygous	21.25	Y	SD	30.29
				BRCA1	Likely	3607C>T (R1203*)	NE	Heterozygous				
68/ 2	Y	N/A	187delAG	*BRCA1*	Likely	68_69delAG (E23fs*17)	NE	Homozygous	23.46	N	SD	5.26
69/ 2	Y	N/A	4184del4	*BRCA1*	Likely	4065_4068delTCAA (N1355fs*10)	NE	NE	NE	Y	SD	9.49
70/ 2	N/A	Y	964del20	*BRCA2*	Likely	736_755delTTTATCGCTTCTGTGACAGA (F246fs*2)	NE	Homozygous	23.8	N	PD	1.48
71/ 2	N/A	Y	1345C>T (Q373X)	*BRCA2*	Likely	1117C>T (Q373*)	NE	NE	9.11	Y	PR	8.34
72/ 2	N/A	Y	8471G>A (G2748D)	*BRCA2*	Known	8243G>A (G2748D)	Germline	NE	NE	Y	PR	7.72
73/ 2	N/A	Y	2986ins4	*BRCA2*	Likely	2758_2759insATGG (P920fs*17)	NE	Homozygous	NE	N	SD	4.30
74/ 2	Y	N/A	IVS19+2delT	*BRCA1*	Likely	5193+2delT (splice site 5193+2delT)	NE	Homozygous	20.52	Y	SD	11.07
75/ 2	N/A	Y	4206ins4	*BRCA2*	Likely	3978_3979insTGCT (A1327fs*4)	Germline	Homozygous	16.49	Y	SD	27.96
76/ 2	N/A	Y	9345G>T (P3039P)							Y	PR	11.14

				Tumor			Tumor SGZ prediction					
Patient/ cohort	g*BRCA1* mutation	g*BRCA2* mutation	g*BRCA* mutation details	*BRCA1*/ *BRCA2*	Pathogenic status	Mutation (protein)	Somatic/ germline	Zygosity	Tumor gLOH (%)	Clinical benefit[1]	Best response[2]	PFS (months)[3]
77/ 2	Y	N/A	2722C>G (S868X)	*BRCA1*	Likely	2603C>G (S868*)	Germline	Homozygous	28.42	Y	PR	5.52
78/ 2	N/A	Y	8803delC	*BRCA2*	Likely	8575delC (Q2859fs*4)	Germline	Homozygous	24.91	Y	PR	16.59
79/ 2	N/A	Y	5301insA	*BRCA1*	Unknown	5252G>A (R1751Q)	Germline	Heterozygous	25.37	N	PD	1.38
				BRCA2	Likely	5073_5074insA (W1692fs*3)	Somatic	Homozygous				
80/ 2	N/A	Y	6252insG							Y	PR	4.17
81/ 2	N/A	Y	6174delT	*BRCA2*	Known	5946delT (S1982fs*22)	NE	Heterozygous	NE	N	SD	5.55
82/ 2	Y	N/A	5083del19	*BRCA1*	Likely	4964_4982delCTGGCCTGACCCCAGAAGA (S1655fs*16)	Germline	Homozygous	36.27	N	SD	9.95
83/ 2	Y	N/A	187delAG							Y	PR	5.55
84/ 2	Y	N/A	2307G>T (E730X)	*BRCA1*	Known	2188G>T (E730*)	Germline	Homozygous	23.88	Y	PR	4.17
				BRCA1	Unknown	2026C>T (P676S)	Germline	Homozygous				

Cohort 1 comprised patients with response to prior platinum and no progression within 8 weeks and Cohort 2 comprised patients who received ≥3 platinum-free cytotoxic regimens.

The test results are from Central laboratories (Myriad BRACAnalysis CDx® for g*BRCA*, FoundationOne® CDx for tumor *BRCA*). Given differences in nucleotide numbering scheme between BRACAnalysis CDx® and FoundationOne® CDx, to assess tumor and germline concordance, mutations were mapped to a common Variation ID in ClinVar (https://www.ncbi.nlm.nih.gov/clinvar/) or similar comparative means were employed.

g*BRCA* mutational status was assessed centrally for 79 patients (using Myriad BRACAnalysis CDx). For the remaining five patients whose samples were not available for central assessment, g*BRCA* mutational status was determined locally for four patients, and the status was unknown for one patient. Tumor *BRCA* variants were assessed using FoundationOne® CDx.

[1]Clinical benefit is based on target, non-target, and new lesions per RECIST 1.1, and confirmation of CR, PR, and SD is not required.

[2]Best response is based on RECIST 1.1 by investigator assessment. Confirmation was required for responses of CR and PR.

[3]PFS is based on RECIST 1.1 by investigator assessment.

Abbreviations: *BRCA*, *breast cancer susceptibility gene*; CR, complete response; gLOH, genomic loss of heterozygosity; ITT, intent-to-treat; N, no; NE, not evaluable; PFS, progression-free survival; PD, progressive disease; PR, partial response; RECIST, Response Evaluation Criteria In Solid Tumors; SD, stable disease; Y, yes.

Supplementary Fig. 1. Study participants

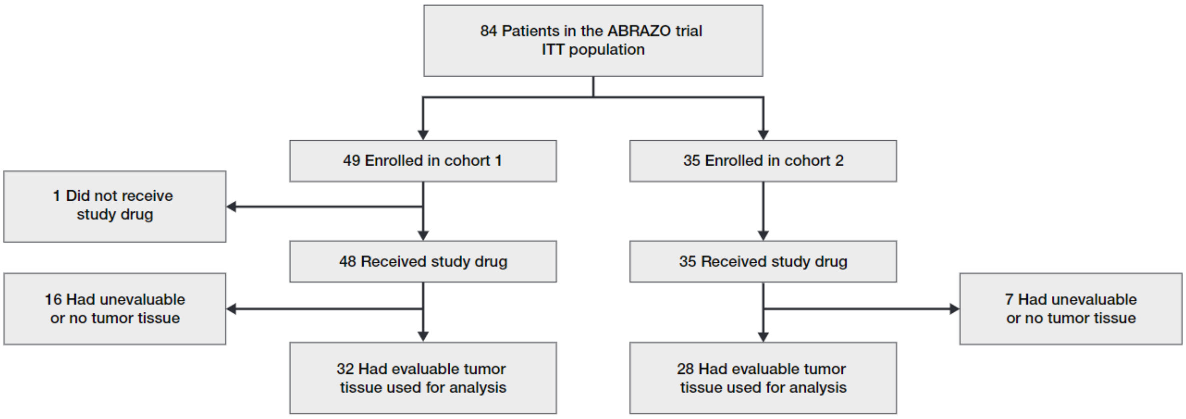

Supplementary Fig. 2. Violin plots comparing gLOH distribution between (a) HER2– and (b) TNBC germline *BRCA* wildtype and germline *BRCA*-mutated tumors in the FoundationCore™ database

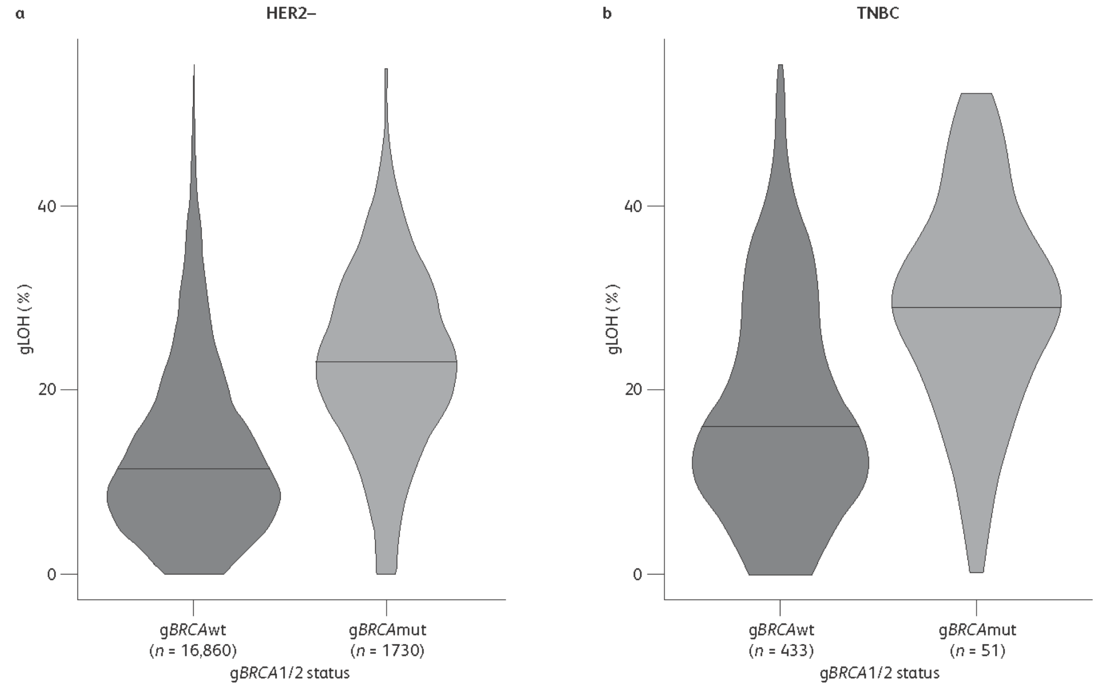

(a) HER2–

p_median[1]	OR_≥16%[2]	p_≥16%[2]
$<2.2\text{x}10^{-308}$	0.13	$<2.2\text{x}10^{-308}$

(b) TNBC

p_median[1]	OR_≥16%[2]	p_≥16%[2]
$7.8\text{x}10^{-10}$	0.16	$2.1\text{x}10^{-7}$

[1]The p value of the Mann–Whitney U test comparing the gLOH values between the two groups.

[2]Odds ratio and p value from Fisher's exact test; statistical comparison is the percentage of samples with gLOH ≥16% between the two groups.

Abbreviations: g*BRCA1/2*, germline *breast cancer susceptibility gene* 1/2; g*BRCA*mut, germline *BRCA* mutation; g*BRCA*wt, germline *BRCA* wildtype; gLOH, germline loss of heterozygosity; HER2–, human epidermal growth factor receptor 2 negative; OR, odds ratio; TNBC, triple-negative breast cancer.
